# The utility of urine sodium–guided diuresis during acute decompensated heart failure

**DOI:** 10.1007/s10741-024-10424-8

**Published:** 2024-08-12

**Authors:** Hasan K. Siddiqi, Zachary L. Cox, Lynne W. Stevenson, Kevin Damman, Jozine M. ter Maaten, Brian Bales, Jin H. Han, Juan B. Ivey-Miranda, JoAnn Lindenfeld, Karen F. Miller, Henry Ooi, Veena S. Rao, Kelly Schlendorf, Alan B. Storrow, Ryan Walsh, Jesse Wrenn, Jeffrey M. Testani, Sean P. Collins

**Affiliations:** 1 Department of Medicine, Vanderbilt University Medical Center, North Tower, 1215 21st Avenue South, 5th Floor, Office 5033C, Nashville, TN 37232-8802 USA; 2 Department of Pharmacy, Lipscomb University College of Pharmacy, Nashville, TN USA; 3 Department of Pharmacy, Vanderbilt University Medical Center, Nashville, TN USA; 4 Department of Cardiology, University Medical Center Groningen, University of Groningen, Groningen, The Netherlands; 5 Department of Emergency Medicine, Vanderbilt University Medical Center, Nashville, TN USA; 6 Geriatric, Research, Education and Clinical Center, Tennessee Valley Healthcare System, Nashville, TN USA; 7 Department of Internal Medicine, Yale University School of Medicine, New Haven, CT USA; 8 Hospital de Cardiologia, Instituto Mexicano del Seguro Social, Mexico City, Mexico; 9 Department of Medicine, Veterans Affairs Tennessee Valley Healthcare System, Nashville, TN USA

**Keywords:** Acute decompensated heart failure, Diuresis, Diuretic strategy, Natriuresis, Decongestion, Urine sodium

## Abstract

Diuresis to achieve decongestion is a central aim of therapy in patients hospitalized for acute decompensated heart failure (ADHF). While multiple approaches have been tried to achieve adequate decongestion rapidly while minimizing adverse effects, no single diuretic strategy has shown superiority, and there is a paucity of data and guidelines to utilize in making these decisions. Observational cohort studies have shown associations between urine sodium excretion and outcomes after hospitalization for ADHF. Urine chemistries (urine sodium ± urine creatinine) may guide diuretic titration during ADHF, and multiple randomized clinical trials have been designed to compare a strategy of urine chemistry–guided diuresis to usual care. This review will summarize current literature for diuretic monitoring and titration strategies, outline evidence gaps, and describe the recently completed and ongoing clinical trials to address these gaps in patients with ADHF with a particular focus on the utility of urine sodium–guided strategies.

## Introduction

Symptoms of congestion are the primary cause of acute decompensated heart failure (ADHF) hospitalizations, and loop diuretics are recommended as first-line therapy to achieve decongestion [1–4]. Although decongestion via diuresis dominates the early management of ADHF [3], there is no well-proven strategy to monitor and titrate diuretic dosing in a safe and efficient manner. This represents an important therapeutic gap as diuretic resistance is common, and at least one-third of patients require diuretic therapy intensification or augmentation during the management of ADHF [5, 6]. In addition, 30–50% of patients discharged from ADHF hospitalization have residual congestion, with worse post-discharge outcomes for those with greater congestion at discharge [7, 8]. A strategy to safely and efficiently achieve euvolemia remains an unmet need.

The goal of decongestion is complicated by challenges in tracking decongestion, assessed by multiple poorly correlated parameters including symptoms and signs of congestion, urine volume, and weight change [8–11]. As a result, a key gap remains in identifying clinical parameters of diuretic response to inform an algorithm guiding effective diuretic titration to achieve optimal decongestion. A net negative sodium balance during diuresis for ADHF has been superior to weight change and urine output for the prediction of post-discharge survival [12, 13]. Thus, urine sodium has emerged as a biomarker of diuretic response that could be incorporated into clinical strategies to guide diuretic titration [4, 14]. We review previous strategy trials of diuretic dosing, current recommendations, and evidence gaps for diuretic titration, including the potential role of urine chemistry studied in recently completed and ongoing clinical trials in ADHF.

## Current evidence for decongestion strategies

### Loop diuretics

Multiple contemporary trials have contributed to current recommendations for initial diuretic dosing and titration [3, 4, 15–21] (Table 1). DOSE-AHF used a 2 × 2 factorial design and randomized 308 hospitalized patients to low- or high-dose furosemide administered by intravenous boluses every 12 h or continuous infusion [16]. The high-dose strategy, starting with an initial intravenous dose of furosemide 2.5 times that of daily oral furosemide equivalents, achieved more fluid loss, weight loss, and symptom relief than the low-dose strategy (total daily IV furosemide dose equivalent to daily outpatient oral loop diuretic dose), and is now one option for an initial diuretic strategy. However, there were no significant differences in the co-primary endpoints of global assessment of symptoms or mean change in creatinine for either of the two dosing strategies or routes of administration [16]. The generalizability of this study is limited by the exclusion of patients admitted with outpatient diuretic dosing exceeding 160 mg furosemide equivalents daily and by the termination of randomized therapy after 72 h.

**Table 1:** Prior decongestion﻿ strategy trials in ADHF

**Trial**	***n***	**Decongestion strategy**	**Diuretic monitoring and titration strategy**	**Primary outcome**	**Limitations to generalization**	**Main result(s)**
EVEREST [15]	4133	1) Tolvaptan + usual care 3) Placebo + usual care	No standardized diuretic strategy	7-day composite: change in GCS and body weight	1) Excluded those with LVEF > 40% 2) Excluded those with SCr > 3.5 mg/dL 3) Enrolled up to 48 h after hospitalization	Greater improvement in 7-day composite outcome with tolvaptan compared to placebo
DOSE [16]	308	1) High-dose diuretic strategy 2) Low-dose diuretic strategy (stratified by bolus vs. continuous infusion)	Standardized initial diuretic dose based on oral dose No standardized strategy to guide dosing change at 48 h or additional open-label diuretic doses	Co-primary at 72 h 1) Patient symptom assessment 2) Change in SCr from 3) baseline	1) Excluded those with SCr > 3.0 mg/d 2) L 3) Physician discretion at 48 h for dosing change or change to open-label 4) Intervention stopped at 72 h	No significant difference in bolus vs. continuous infusion or high-dose vs. low-dose for patient-reported symptoms or change in creatinine at 72 h
CARRESS [17]	188	1) Stepped pharmacologic 2) therapy 3) Ultrafiltration	Standardized diuretic titration based on urine output volume	Bivariate change in 2 outcomes at 96 h: 1) Creatinine 2) Body weight	1) Only enrolled those with WRF but excluded those with SCr > 3.5 mg/dL 2) Open-label design 3) Intervention stopped at 96 h	Ultrafiltration inferior to stepped pharmacologic therapy at 96 h for creatinine and body weight
ROSE-AHF [18]	360	1) Low-dose dopamine 2) Low-dose nesiritide 3) Usual care	Standardized initial diuretic dose based on oral dose No standardized strategy to guide diuretic dosing change starting at 24 h	Co-primary at 72 h: changes in urine volume and cystatin-C	1) Excluded patients with eGFR > 60 or < 15 mL/min/m^2^ and anticipated hospital stay > 72 h 2) Intervention stopped at 72 h	No difference at 72 h from usual care for either dopamine or nesiritide group on change in urine volume or cystatin
3T Trial [19]	60	Standardized loop diuretics plus either: 1) Oral metolazone 2) IV chlorothiazide 3) Tolvaptan	Standardized diuretic titration based on urine output volume	Weight loss at 48 h	1) Only enrolled patients with diuretic resistance 2) Intervention stopped at 48 h 3) Excluded patients with eGFR < 15 mL/min/m^2^	Similar weight loss in all three groups without a statistically detectable between group difference
ADVOR [20]	519	Standardized loop diuretics plus either: 1) Acetazolamide 2) Placebo	Standardized diuretic titration based on urine output volume	Achievement of decongestion at 72 h by blinded investigator clinical assessment	1) Excluded patients requiring IV furosemide doses > 80 mg prior to enrollment 2) Excluded patients on SGLT2i 3) Intervention stopped at 72 h 4) Excluded patients with eGFR < 20 mL/min/m^2^	Significantly greater successful decongestion in acetazolamide group compared to placebo No difference in all-cause mortality or HF hospitalization at 3 months between groups
CLOROTIC [21]	230	Standardized loop diuretics plus either: 1) Hydrochlorothiazide 2) Placebo	Standardized initial diuretic dose based on oral dose No standardized strategy to guide diuretic dosing change starting at 24 h	Co-primary endpoints: 1) Change in body weight at 72 h compared to baseline 2) Patient-reported dyspnea at 72 h compared to baseline	1) Lower than anticipated enrollment 2) Unbalanced baseline characteristics between groups 3) Did not specify degree of congestion/volume overload needed for inclusion 4) Long-term renal function not monitored 5) Intervention stopped at 5 days	Statistically greater weight loss, but no difference in patient-reported dyspnea at 72 h in HCTZ group compared to placebo No difference in mortality or hospitalization

*GCS* global clinical status, *WRF* worsening renal function, *LVEF* left ventricular ejection fraction, *IV* intravenous, *SCr* serum creatinine, *eGFR* estimated glomerular filtration rate, *SGLT2i* sodium-glucose co-transporter 2 inhibitors

### Ultrafiltration

Ultrafiltration (UF) removes plasma volume at a set rate across a membrane using a pressure gradient, with encouraging results in early studies [22–24]. In the CARRESS-HF trial, 188 patients hospitalized for ADHF with worsening renal function and signs of persistent congestion were randomized to pharmacologic therapy with a suggested algorithm of diuretic escalation based on daily urine volume and persistent congestion versus UF [17]. In the stepped pharmacologic therapy arm, escalations were recommended based on daily urine volume and the persistence of clinical congestion. The trial was stopped early for the inferiority of the UF strategy as defined by the bivariate endpoint of absolute change in weight from randomization to 96 h and absolute change in serum creatinine (*p* = 0.003). This was primarily due to a progressive increase in the serum creatinine in the UF arm over the first 96 h of the study, an effect now considered associated with a diuretic response and often tolerated without changes in therapy during the decongestion phase of ADHF [11, 12]. Fewer than 10% of patients in either group achieved decongestion by the end of the study, but the UF arm had a significantly higher rate of adverse events (primarily more kidney failure, bleeding, and infectious complications as well as catheter-related complications) at 60 days [17]. Subsequent studies of UF had inadequate enrollment and early termination [25]. Given the results of CARRESS-HF, the routine use of UF for decongestion in ADHF is not a guideline recommendation [1].

### Diuretic combinations of loop and non-loop diuretics

Inadequate response to loop diuretics despite dose escalation often leads to the addition of another diuretic agent. While the addition of thiazide or thiazide-like diuretics is currently recommended, there is limited evidence regarding the use of adjunctive diuretics [2–4, 14]. The 3T trial in ADHF found no difference between IV chlorothiazide and oral metolazone in patients who exhibited diuretic resistance despite high-loop diuretic doses [19]. However, due to the lack of a control group receiving loop diuretic monotherapy only, the benefit of these additional therapies on top of loop diuretics could not be determined in this trial. The use of hydrochlorothiazide (HCTZ) was studied in 230 patients admitted with ADHF who were randomized to the addition of oral HCTZ or placebo to a protocolized furosemide diuretic regimen in the CLOROTIC trial [21]. Adjunctive HCTZ resulted in greater weight loss at 72 and 96 h, but also more hypokalemia and renal dysfunction, particularly in women. However, weight loss between groups was not significantly different at discharge, a convergence which may be anticipated in such a trial as other diuretics may be escalated and optimized for relief of symptoms and signs of congestion after the 96-h study treatment window ended [21, 26].

Other diuretic combinations have recently been investigated. While mineralocorticoid receptor antagonists are an important part of guideline-directed medical therapy for chronic HF with reduced ejection fraction, high-dose spironolactone did not show any symptomatic or clinical benefit atop standard ADHF therapies among the 360 hospitalized patients enrolled in the ATHENA-HF trial [27]. This null effect could be due to spironolactone’s pharmacokinetics causing delayed onset of action, aldosterone-independent activation of the epithelial sodium channel via urinary plasmin, or absence of a diuretic-resistant state [27–30]. The use of acetazolamide to augment diuresis in ADHF was studied in the ADVOR trial of 519 patients randomized to protocolized loop diuretic therapy with the addition of acetazolamide 500 mg IV or placebo once daily [20]. Acetazolamide use resulted in significantly greater freedom from congestion within 3 days of randomization compared to the control group (primary endpoint), with a similar incidence of renal dysfunction, hypokalemia, and hypotension and no difference in all-cause mortality or rehospitalization for heart failure. Added to the median dose of 120 mg/day intravenous furosemide, acetazolamide resulted in a modest median increase of 0.5 L [95% CI 0.2–0.8 L] urine output and 98 mmol [95% CI 56–140] of urine sodium at 48 h [20]. Importantly, the concurrent use of sodium-glucose cotransporter 2 inhibitors (SGLT2i) was an exclusion for this trial. As a result, the clinical impact of this strategy when compared to escalating loop diuretic dosing and in patients receiving SGLT2i needs further assessment.

### Additional therapies to improve congestion

The advent of SGLT2i is of increasing interest as an adjunct to diuresis [31, 32]. Mechanistic studies of SGLT2i show increased natriuresis and diuresis, an effect that is synergistic when added to loop diuretics [33, 34]. However, several factors including counter-regulatory and anti-natriuretic mechanisms that are activated with SGLT2 inhibition also play a key role in determining the true natriuretic/diuretic effect of SGLT2i in each patient [34]. The EMPAG-HF trial randomized 60 patients admitted to the hospital with ADHF to either regular diuretic therapy or the addition of empagliflozin 25 mg daily on top of standard therapies [35]. The results showed a 2.2-L increase in cumulative urine output over 5 days for the SGLT2i group compared to the standard of care group, without a worsening of safety and renal outcomes. These results were similar to the EMPA-RESPONSE-AHF trial, lending further credence to the use of SGLT2i in this setting [36] The DICTATE-AHF trial further studied this question among 240 patients hospitalized for ADHF. Patients were randomized to either dapagliflozin 10 mg daily or usual care in addition to a standardized loop diuretic treatment algorithm within 24 h of presentation [31]. There was no difference in the two groups for diuretic efficiency (cumulative weight change per cumulative loop diuretic dose), but dapagliflozin use was associated with lower loop diuretic doses and fewer diuretic up-titrations during the hospitalization, without any difference in renal or cardiovascular safety endpoints [37]. These results suggest that early initiation of SGLT2i is safe and may help with diuresis, while also providing an opportunity to optimize guideline directed medical therapy during the hospitalization.

Similarly, the use of the vasopressin antagonist, tolvaptan, has been shown to be safe and effective overall in ADHF. In the TACTICS-HF trial, 257 patients were randomized within 24 h of admission for ADHF to either 30 mg daily of tolvaptan or placebo in addition to conventional diuretic treatment [38]. There was a significantly higher amount of urine output and weight loss in the tolvaptan group compared to placebo, although this was associated with worsening renal function. Additional trials in vasopressin antagonists have not shown a clear benefit in this population; thus, the role of vasopressin antagonists in ADHF remains limited [38, 39]. Other therapies such as nesiritide, serelaxin, dopamine, and rolofylline have been investigated in hopes of improving outcomes both during and after hospitalization for ADHF [18, 40–43]. None of these agents has been shown to have consistent and significant clinical benefit.

## Guidelines on diuretic therapy and titration

Current international HF guidelines and expert panels recommend diuretic titration to alleviate congestion without strong recommendations for diuretic monitoring parameters to guide diuretic therapy titration [1, 2, 4] (Table 2). The 2019 ACC Expert Consensus Decision Pathway for HF hospitalization maps daily assessment of the trajectory toward decongestion for hospitalized patients, including optional use of adjunctive diuretics for patients in whom diuresis has not been effective or has stalled after early response [3]. The 2022 AHA/ACC/HFSA guidelines also endorse careful tracking of fluid input and output measures, vital signs, daily standing weights, and the signs and symptoms of congestion and hypoperfusion [1]. However, without the inclusion of biomarkers or objective metrics to guide diuretic therapy, there remains great ambiguity and variability in how to achieve the desired decongestion.

**Table 2:** Guideline recommendations for diuretic titration and intensification in ADHF

**COR**	**LOE**	**Recommendations**
**2022 ACC/AHA/HFSA Guideline for the Management of Heart Failure**		
**2a**	**B-NR**	In patients hospitalized with HF when diuresis is inadequate to relieve symptoms and signs of congestion, it is reasonable to intensify the diuretic regimen using either (a) higher doses of intravenous loop diuretics or (b) addition of a second diuretic
**2021 ESC Guidelines for the diagnosis and treatment of acute and chronic heart failure**		
**2a**	**B**	A combination of a loop diuretic with a thiazide-type diuretic should be considered in patients with resistant edema who do not respond to an increase in loop diuretic doses

*COR* class of recommendation, *LOE* level of evidence

The European Society of Cardiology 2021 guidelines provide more specific recommendations for monitoring diuretic response, while also emphasizing the limited evidence for optimal dosing and titration of diuretics [2]. Citing observational data and expert panel advice, the ESC guidelines advocate checking a spot urine sodium concentration 2 h after diuretic dosing, in addition to measuring hourly urine output [4, 13]. A urine output of greater than 100–150 mL/h for the first 6 h or a spot urine sodium concentration of greater than 50–70 mEq/L at 2 h post diuretic dosing is suggested as an appropriate diuretic response, with continuation of current diuretic dosing if these criteria are met. This guideline suggests those patients who do not achieve this level of response should have a doubling of the IV diuretic dose initially, followed by the addition of non-loop diuretics if the diuretic response remains unfavorable [4, 13]. All the guidelines emphasize the opportunity for guideline-directed medical therapy optimization during an ADHF hospitalization as a goal, particularly with SGLT2i and mineralocorticoid receptor antagonist therapy. The timing of GDMT initiation and optimization in relationship to decongestive therapy and diuresis is a topic of ongoing discussion and merits further investigation.

## Challenges, evidence gaps, and an unmet need for clinical trials

**Fig. 1:**
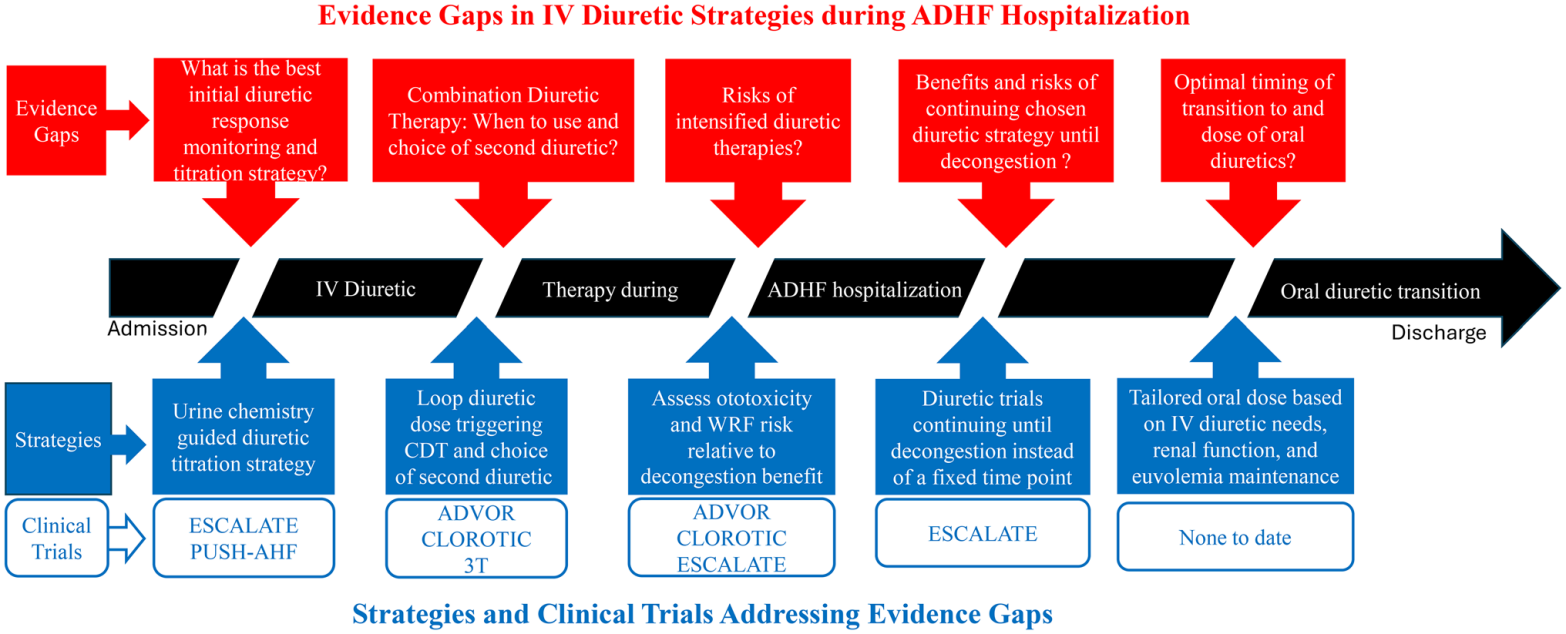
Evidence gaps, potential strategies, and ongoing clinical trials to determine optimal strategies for IV diuretic titration during acute decompensated heart failure hospitalizations. There remain many evidence gaps in determining optimal IV diuretic strategies during acute decompensated heart failure hospitalizations to relieve congestion. Several proposed strategies are outlined, with notation of the clinical trials that are currently underway to test these strategies. IV, intravenous; CDT, combination diuretic therapy; WRF, worsening renal function

While there is general agreement on entry criteria for ADHF trials and admission diuretic dosing, prior trial limitations have resulted in evidence gaps for best practices in diuretic use, titration, and transitions that require further investigation (Fig. 1). Key evidence gaps include (1) establishing volume status and characterizing degrees of congestion and “euvolemia,” (2) identifying the best strategies to monitor diuretic response/resistance and titrating diuretic therapy to patient volume status, (3) balancing the expected increases in serum creatinine levels with goals for complete decongestion, (4) delineating risks of intensified diuretic strategies, including electrolyte abnormalities and ototoxicity risk, (5) understanding the efficacy of combination diuretic therapy, including the optimal dose of loop diuretic before adding alternative agents, as well as the choice of a second or third diuretic agent, (6) extrapolating diuretic strategies for the entire ADHF hospitalization from current trial data which often limit interventions to the first 48 to 72 h of decongestion, and (7) optimal timing and choice/dosing of oral diuretic transition along with post-hospitalization volume management. In addition to the need to address these evidence gaps, clinical trials in this space also face the challenge of utilizing outcomes reflective of the multiple components of ADHF, including the patient’s perspective, and heterogeneity of response due to baseline HF co-morbidities. Composite and co-primary outcomes are often necessary to capture the multi-dimensional nature of this complicated topic. Furthermore, there are several different disease states and physiologies driving ADHF, and different patients may have different target volume statuses and thus different decongestion goals. Finally, the distribution of fluid between the extravascular and intravascular compartments and the refill rate during net diuresis is a key process in effective diuresis, and another knowledge gap meriting further investigation.

As upcoming diuretic trials address these evidence gaps, it will be important to achieve a balance regarding the scientific rigor of strategy trials and the feasibility of translating the winning strategy into clinical settings without dedicated research staff. If a strategy is found to be more effective than usual care, yet overly burdensome to implement, the impact on clinical care for ADHF may be limited.

## Potential role of and evidence for urine chemistry-driven decongestion strategies

There is emerging interest in urine chemistry guidance for diuretic monitoring and titration strategies. Urine sodium concentration has been evaluated in multiple observational ADHF cohort studies [13, 44–51] (Table 3). Lower urine sodium concentrations following the first IV loop diuretic dose are associated with worse prognoses, including worsening kidney function, worsening HF, and mortality [13, 47, 48, 50–53]. Based on the urine sodium concentrations observed in these cohorts, experts have suggested measuring the urine sodium concentration 1–2 h after the initial diuretic dose and titrating the diuretic dose to achieve a spot urine sodium concentration > 50–70 mmol/L [2, 4, 14]. The reason for this early assessment is twofold: first, it allows for early detection of diuretic response, and second, it is thought that in most patients the peak diuretic effect is achieved approximately 1 to 2 h after IV dosing. However, causality cannot be assumed between increased natriuresis and improved outcomes. Recent trials have investigated this relationship, and it is a central goal of the ongoing ESCALATE trial which is described below in detail [54, 55]. A current strategic question is whether and how to incorporate urine sodium and natriuresis in guiding diuretic titration and decongestion.

**Table 3:** Prospective inpatient studies evaluating urine sodium concentration

**Study name (citation)**	**Use of urine chemistry**	**Time of urine collection**	***n***	**Primary outcome(s)**	**Randomized/** **blinded**	**Comparator group**
Singh et al. 2014 [44]	Spot UNa, UCr, urine furosemide level	Spot urine during continuous furosemide infusion	52	Natriuretic response, diuresis, and clinical outcomes	No/no	None
Ferreira et al. 2016 [45]	Spot UNa, UK	Day 1 and 3	100	Cardiovascular mortality and/or ADHF hospitalization at 180 days	No/no	Spironolactone vs. no spironolactone
Testani et al. 2016 [46]	Spot UNa, UCr for modified NRPE calculation	Spot urine 2 h after IV diuretic	50	Sodium output after IV diuretic dose predicted by spot urine chemistry	No/no	None
Luk et al. 2018 [47]	UNa	First voided urine after IV diuretic dose	103	Composite of placement of MCS device during hospital stay, discharge from hospital on inotropes, or death at admission or during the follow-up period	No/no	None
Honda et al. 2018 [48]	UNa	At the time of hospital admission	669	Composite of all-cause death and WHF	No/no	Tertiles of admission UNa
Cunningham et al. 2019 [49]	UNa	Cumulative urine in first 24 h after randomization	298	Length of stay	No/no	UNa ≤ 60 mmol/L vs. > 60 mmol/L
Collins et al. 2019 [50]	UNa	One h after initial diuretic	61	WHF	No/no	None
Biegus et al. 2019 [51]	UNa	Prior to IV diuretic, 6, 24 and 48 h	111	All-cause mortality at 1 year	No/no	UNa response at 6 and 48 h
Damman et al. 2020 [13]	UNa	6 and 24 h	175	Urine volume and all-cause mortality	No/no	None

*UNa* urine sodium, *UCr* urine creatinine, *UK* urine potassium, *WHF* worsening heart failure, *NRPE* natriuretic response prediction equation

Two recent clinical trials have shed further light on the utility of urine sodium measurement to aid decongestion for ADHF. Pragmatic Urinary Sodium-based treatment algoritHm in Acute Heart Failure (PUSH-AHF) was a single-center, open-label, randomized, pragmatic strategy trial designed to test the hypothesis that a low concentration of spot urine sodium after diuretics can be utilized to inform diuretic dosing and maximize natriuresis and diuresis [56, 57]. PUSH-AHF randomized 310 patients presenting to the emergency department with ADHF requiring IV diuretics to (1) usual care vs. (2) urinary sodium–guided diuretic therapy using a urinary sodium threshold of < 70 mmol/L (and diuresis < 150 mL/h at later timepoints) to inform the subsequent diuretic dose [56] (Table 4). All urinary analyses, including natriuresis, in the usual care arm were blinded until study completion. Patients randomized to the intervention arm had spot urine sodium checked 2 h after the initial dose of IV diuretic, and then at 6, 12, 18, 24, and 36 h. If the spot urine sodium was < 70 mmol/L or diuresis < 150 mL/h at hour 6 onwards, then patients had their diuretic dose doubled to a maximum of 5 mg of bumetanide twice daily, and if this was not effective, then HCTZ was added followed by either acetazolamide or an SGLT2i. At 2, 6, 12, 18, 24, and 36 h, doses were adjusted per the above algorithm, and the dose that achieved urine sodium or diuretic goal was scheduled twice daily until 48 h after randomization. After 48 h, the adjustment of decongestive therapy was left to the discretion of the treating team [57].

PUSH-AHF’s co-primary outcomes were a total of 24-h natriuresis as well as the first occurrence of all-cause mortality or HF rehospitalization at 6 months [56]. Patients in the natriuresis-guided group had significantly greater mean total natriuresis at 24 (*p* = 0.0061) and 48 h (*p* = 0.0241); however, this difference was not found at 72 h. Changes in NT-proBNP by 24 and 48 h and the safety outcomes including renal function were the same in both arms, as was length of stay and all-cause mortality or HF rehospitalization at 180 days [56]. This trial showed the effectiveness and safety of a natriuresis-guided diuretic strategy to increase natriuresis and diuresis in the first 48 h but the lack of sustained benefit may be in part due to the end of randomized therapy after the initial 48 h.

The Efficacy of a Standardized Diuretic Protocol in Acute Heart Failure (ENACT-HF) study was a multicenter, non-randomized, open-label two-phase study of 401 patients with ADHF hospitalized in 18 countries comparing standard care at each center with a standardized diuretic titration protocol [58, 59]. In the first phase, patients were treated per each institution’s standard care with no guidance from the study team. In the second phase, a standardized diuretic protocol derived from the Heart Failure Association position paper was used at each center for diuretic titration [4]. Loop diuretic intravenous boluses were given twice daily, with the initial dose given twice the oral maintenance dose with a maximum of 200 mg furosemide equivalents per dose. A spot of urine sodium was collected after 2 h, and diuretic response was judged based on the urine sodium (goal > 50 mmol/L) and urine output (goal > 100 mL/h). If either of these conditions were not met after the first dose, the next dose would be doubled followed by the addition of a thiazide diuretic. If the daily urine output after 24 h was less than 3000 mL, then the diuretic regimen was escalated following an identical titration algorithm. The diuretic protocol was used for 2 consecutive days, after which patients returned to treatment at the discretion of the local treating physician. ENACT-HF’s primary outcome was natriuresis after 1 day, with secondary endpoints including a variety of clinical outcomes [58]. Patients in the protocol arm had significantly greater natriuresis after 1 (*p* < 0.001) and 2 days (*p* < 0.001) compared to those in the standard-of-care arm. There was also significantly greater diuresis after 2 days in the protocol arm compared to the standard-of-care arm (*p* < 0.001). Hospital length of stay was significantly shorter in the protocol arm (5.8 days vs. 7.0 days, *p* = 0.036), without a significant difference between groups for in-hospital mortality or safety outcomes [58].

**Table 4:** Clinical trials of urine chemistry-guided diuresis

**Trial characteristic**	**ENACT-HF** [58]	**PUSH-AHF** [56]	**ESCALATE** [55]
Maximum dose of IV diuretic	200 mg IV furosemide twice daily	25 mg IV bumetanide in first 24 h, then 5 mg IV bumetanide twice daily for remainder of trial	500 mg IV furosemide thrice daily
Diuretic augmentation (if needed)	Thiazide	Oral hydrochlorothiazide. Second line options are IV acetazolamide or SGLT2i	IV chlorothiazide
Goal of diuretic titration	Spot urine sodium > 50 mmol/L and diuresis > 100 mL/h	Spot urine sodium ≥ 70 mmol/L or diuresis ≥ 150 mL/h	Achieving total natriuresis goal set by treating clinical team, utilizing NRPE for natriuresis estimate
Randomized	No	Yes	Yes
Placebo utilized	No	No	Yes
Blinded allocation	No	No	Double-blind
Participants	401	310	450
Duration of intervention	48 h	48 h	Duration of IV diuretic use during hospitalization
Primary endpoint	Natriuresis after 1 day	Co-primary endpoints: total 24-h natriuresis, 6-month all-cause mortality/HF hospitalization	Net clinical benefit over 14 days (incorporates time on IV diuretic, clinical status, patient symptoms)
Pragmatic design	Yes	Yes	No

*HF* heart failure, *SGLT2i* sodium-glucose co-transporter 2 inhibitors, *IV* intravenous

## Using the natriuretic response prediction equation to guide diuresis

The natriuretic response prediction equation (NRPE) incorporates urine sodium concentration, renal function, and body size to estimate the 6-h natriuretic response, which can then be used to titrate diuretics as diuresis goals are closely linked to the natriuretic response. The NRPE predicts natriuresis in response to diuretics with greater accuracy than urine sodium concentration alone [46]. The goal of the NRPE is to inform diuretic strategies and accelerate the achievement of net negative sodium balance during ADHF [8]. The NRPE, as seen in the formula below, calculates 6-h sodium excretion by estimating the instantaneous rate of urine production as the product of the estimated glomerular filtration rate (eGFR) and the ratio of serum to urine creatinine and converting this value into cumulative sodium excretion using the urinary sodium concentration and a constant [61] (38).

$$\mathrm{Na}\;\mathrm{output}\;\left(\mathrm{mmol}\right)=\mathrm{eGFR}\times\left(\mathrm{BSA}/1.73\right)\times\left({\mathrm{Cr}}_{\mathrm{serum}}/{\mathrm{Cr}}_{\mathrm{urine}}\right)\times60\;\mathrm{minutes}\times3.25\;\mathrm{hours}\times\left({\mathrm{Na}}_{\mathrm{urine}}/1000\mathrm{ml}\right)$$

Na = sodium, eGFR = estimated glomerular filtration rate, BSA = body surface area,

Cr_Serum_ = serum creatinine, Cr_Urine_ = urine creatinine, Na_Urine_ = urinary sodium concentration.

The NRPE has been validated against a measured 6-h cumulative sodium excretion, demonstrating excellent discrimination across a range of natriuretic responses (area under the curve $\ge$ 0.90) [46, 61]

**Fig. 2:**
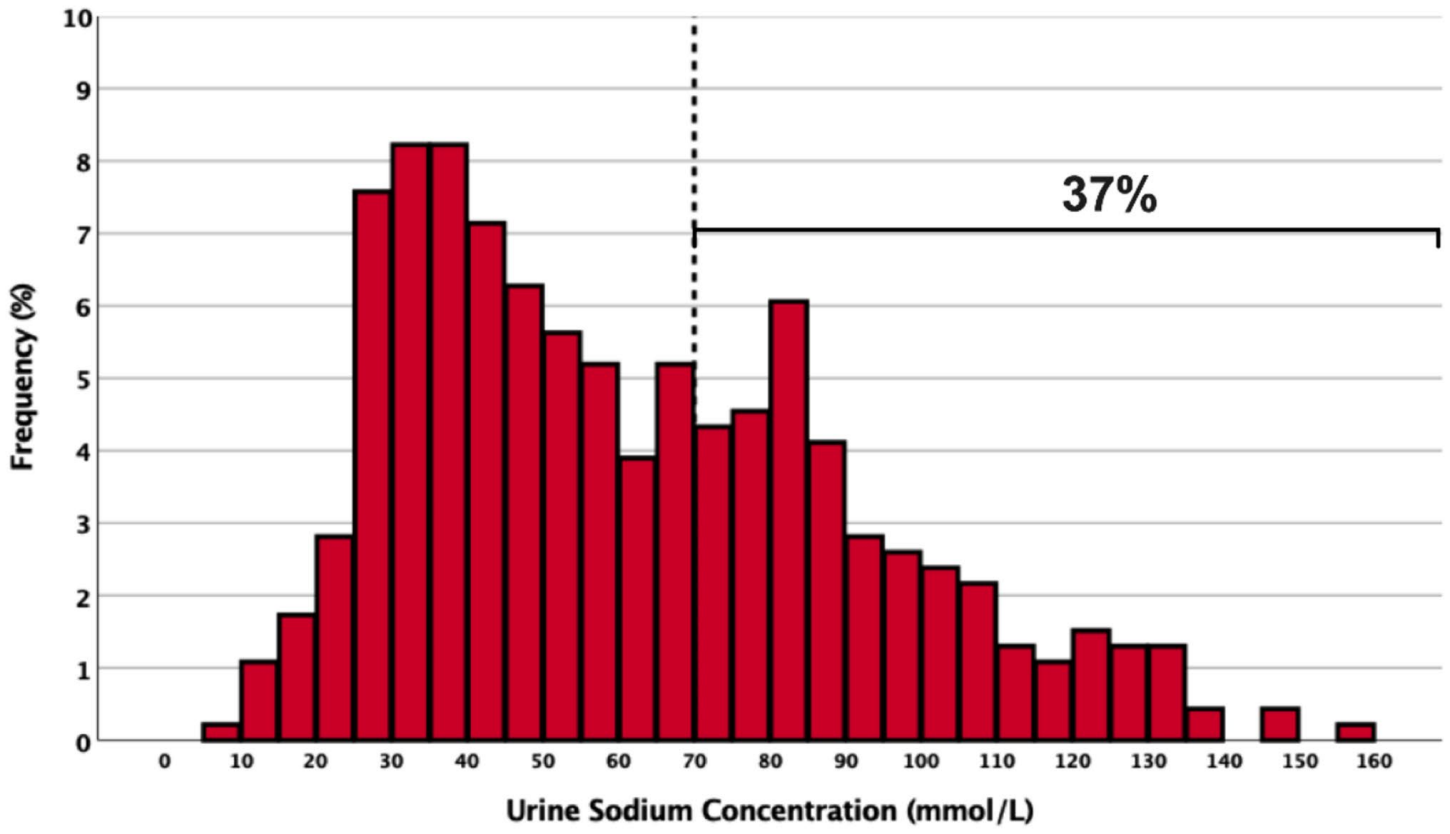
The urine sodium concentration (*n*=462) from urine samples collected immediately before the intravenous (IV) loop diuretic dose was administered in 285 patients with ADHF undergoing serial IV diuretic doses is presented as a histogram. These results suggest that 37% of patients would be categorized as diuretic “responders” *prior to* receiving an IV diuretic if a urine sodium concentration threshold of 70 mmol/L is used to identify responders. Data adapted from reference [60]

**Fig. 3:**
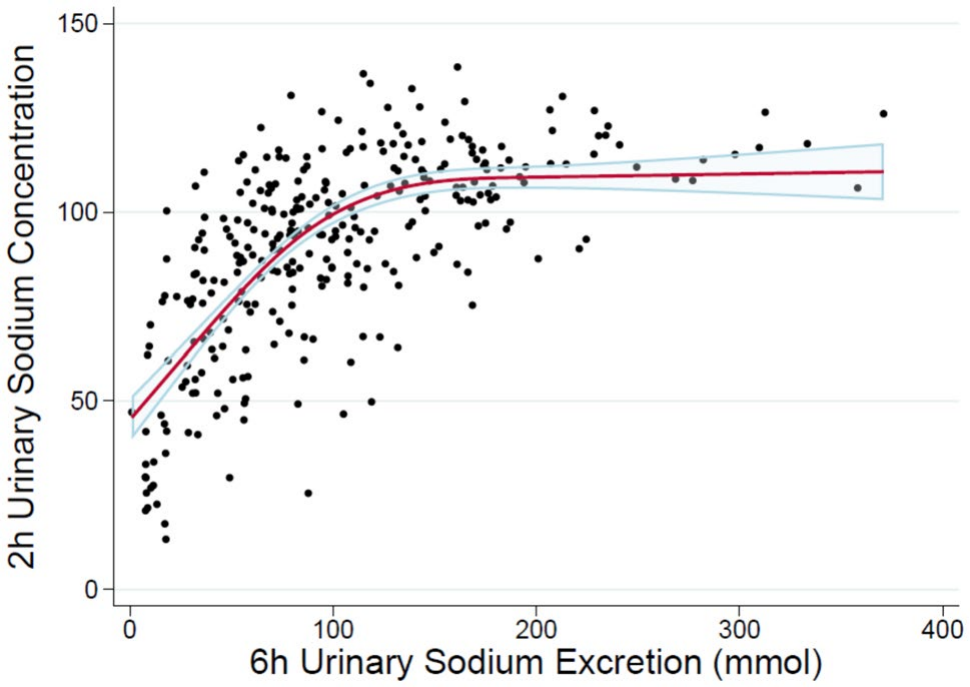
Non-linear association between spot urine sodium concentration 2 hours after diuretic dosing and total urinary sodium excretion at 6 hours. At lower values, 2-h spot urine sodium may be linear with 6-h urine sodium excretion. At lower values of 2-h spot urine sodium the relationship with 6-h urinary sodium excretion may be linear. However, this relationship appears to not be linear at higher 2-h urinary sodium concentrations, thereby making it harder to predict natriuresis from urine sodium alone. Data adapted from reference [61]

The NRPE was also shown to inform diuretic dosing to improve congestion in an observational study [61]. Understanding the differences in efficacy and safety between a strategy of spot urine sodium titration compared with titration using the NRPE will be important to understand. Spot urine sodium concentration is a more straightforward method of quantifying response, allowing the clinical team to titrate diuretics in a binary way based on urine sodium concentration (i.e., spot urine sodium < or ≥ 70 mEq/L). However, spot urine sodium measurements can be misleading if not interpreted in relation to the urine concentration. Using urine sodium alone may falsely reassure the provider of an adequate natriuretic response when the urine is concentrated despite having urine sodium output that is less than the goal, a phenomenon seen in up to 37% of patients (Fig. 2). Therefore, using urine sodium alone may be less accurate in predicting diuretic-induced natriuresis (Fig. 3). By utilizing both urine sodium and creatinine, urine dilution is accounted for in the NRPE. However, there are limitations to the use of the NRPE. Utilization of the NRPE is more cumbersome than the use of a binary spot urine sodium value, and this may impede its uptake into clinical practice. In addition to requiring spot urine creatinine and sodium as well as serum creatinine values, the NRPE requires access to an online calculator to quantify diuretic response and inform subsequent dosing recommendations.

### ESCALATE

The urinE chemiStry guided aCute heArt faiLure treATmEnt (ESCALATE; U01HL084877) trial is a randomized controlled double-blinded strategy trial ongoing at two academic hospitals in the United States [55] (Table 4) testing the hypothesis that protocolized diuretic therapy guided by spot urine chemistry using the NRPE will be superior to usual care at improving outcomes over the 14 days following randomization. This trial will determine if the clinical benefits of a net negative sodium balance seen in observational studies translate into efficacy and safety as a diuretic strategy. The investigators aim to randomize 450 patients with ADHF to (1) usual care vs. (2) NRPE-driven care. Once randomized, patients receive an initial open-label IV furosemide bolus to initiate the protocol, with standard initial dose recommendations based on prior diuretic use and guideline recommendations [1, 16]. In both arms, the patients’ clinical team sets a daily goal for diuresis as well as their estimation of the patients’ global volume assessment (Table 5). Patients randomized to the usual care arm have diuretic dosing determined by the clinical team. In patients randomized to the intervention arm, urine sodium and creatinine results are entered into the NRPE, and a dose of IV furosemide (and additional thiazide if called for) is determined based on the natriuretic response to the prior dose of diuretic and relative achievement of the clinical team’s desired daily net-negative diuresis goal. The maximum doses of diuretics in the intervention arm are 500 mg IV furosemide three times daily with or without 500 mg IV chlorothiazide once a day. Unlike prior diuretic strategy trials which stopped at pre-determined timepoints, the diuretic strategies in both arms are continued until the patient transitions from IV to oral diuretics as determined by the clinical team, therefore allowing for a more complete understanding of the strategy’s benefits and risks.

The primary outcome in ESCALATE is the number of days of net clinical benefit between the treatment and control arms during the 14 days after randomization [55]. Net clinical benefit is calculated in this study by integrating a score representing each of three important components of a patient’s HF hospitalization: (1) daily clinical state (IV diuretics vs. oral diuretics, admitted vs. discharged, alive vs. deceased), (2) symptoms (reported daily by patients on a visual analogue scale), and (3) duration in each of these states/symptoms. The number of days of net clinical benefit will be expressed as the mean difference in days between the treatment arms where being in one treatment arm was of greater benefit than being in the other treatment arm. This study aims to be completed in 2026.

**Table 5:**
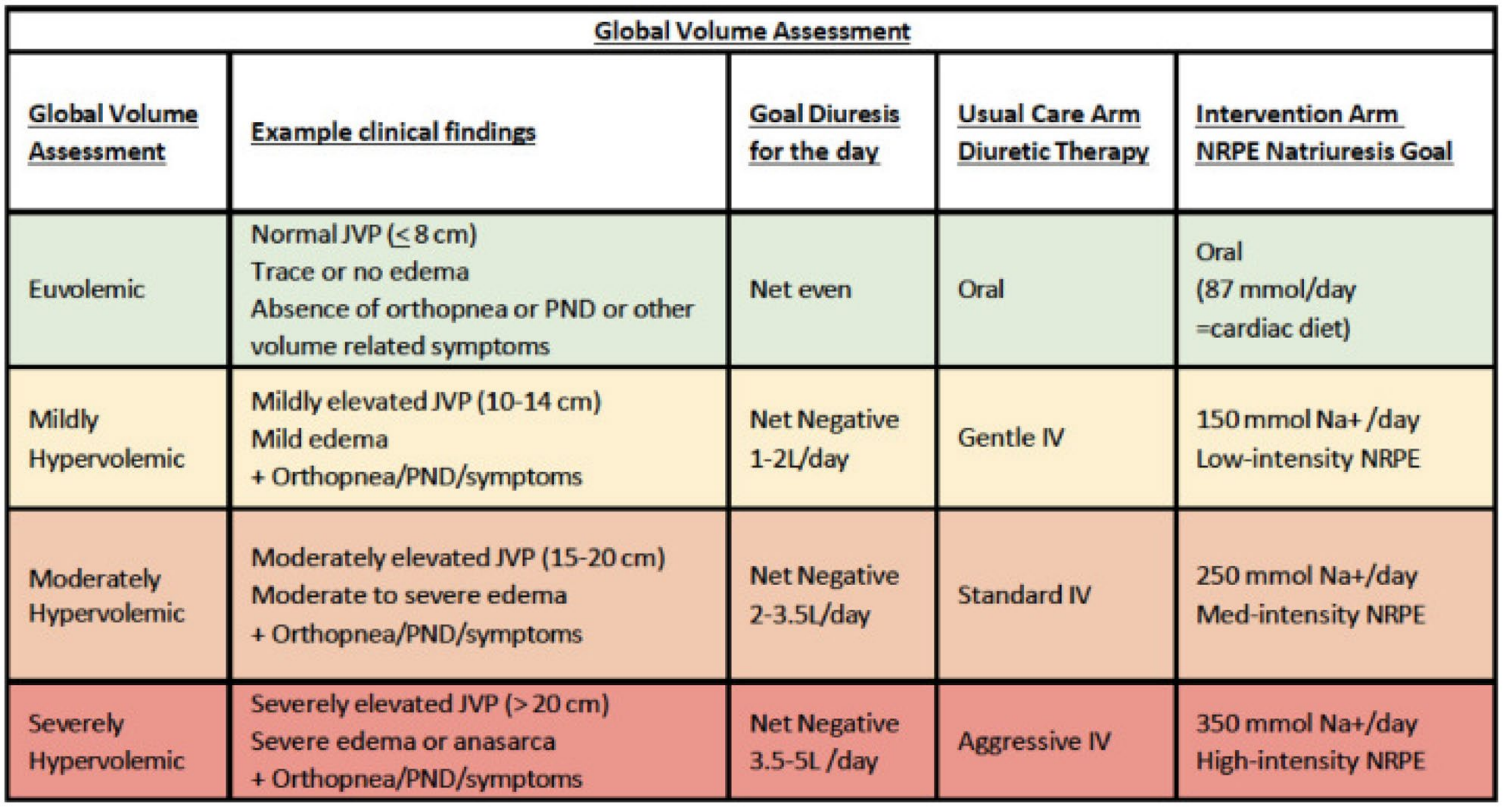
Daily structured volume assessment and decongestive goals in the ESCALATE trial

*cm* centimeters of H_2_0, *IV* intravenous, *JVP* jugular venous pressure, *L* liters, *mmol* millimole, *Na*^+^ sodium, *NRPE* natriuretic response prediction equation, *PND* paroxysmal nocturnal dyspnea

## Conclusions

Hospitalizations for ADHF represent a major burden on patient quality of life, cost of medical care, morbidity, and mortality. While decongestion is the cornerstone of ADHF management, there is currently no robust and reproducible evidence-based method of achieving this goal. Measurement and diuretic titration utilizing urine sodium (including the NRPE) has shown promise as a method of characterizing individual diuretic responsiveness. Recent and ongoing trials will provide additional information on the safety and efficacy of a urine sodium–guided approach to inpatient diuresis and, if they demonstrate efficacy, will facilitate personalized diuretic therapy to achieve decongestion in the many patients hospitalized with ADHF.

## Data Availability

All data supporting this figure are available within the cited paper and its Supplementary Information.

## Abbreviations

ADHF: Acute decompensated heart failure
IV: Intravenous
UF: Ultrafiltration
SGLT2i: Sodium-glucose co-transporter 2 inhibitor
HF: Heart failure
NRPE: Natriuretic response prediction equation
HCTZ: Hydrochlorothiazide
